# The simultaneous expression of both ephrin B3 receptor and E-cadherin in Barrett`s adenocarcinoma is associated with favorable clinical staging

**DOI:** 10.1186/2047-783X-17-10

**Published:** 2012-05-14

**Authors:** Matthias C Schauer, Nikolas H Stoecklein, Joerg Theisen, Feride Kröpil, Stephan Baldus, Arnulf Hoelscher, Markus Feith, Edwin Bölke, Christiane Matuschek, Wilfried Budach, Wolfram Trudo Knoefel

**Affiliations:** 1 Department of General-, Visceral-, and Pediatric Surgery, Heinrich Heine Universitaet Duesseldorf, Duesseldorf, Universitätsstraße, 140225, Düsseldorf, Germany; 2 Department of Pathology, Heinrich Heine Universitaet, Duesseldorf, Germany; 3 General-, Visceral-, and Oncological Surgery, Universitaetsklinikum Koeln, Koeln, Germany; 4 Department of Surgery, Technische Universitaet Muenchen, Munich, Germany; 5 Department of Radiotherapy, Heinrich Heine University, Duesseldorf, Germany

**Keywords:** E-cadherin, Ephrin B3 receptor, Esophageal adenocarcinoma, Barrett’s metaplasia

## Abstract

**Background:**

In intestinal epithelium, tyrosine kinase receptor Ephrin B3 (Eph B3) maintains the architecture of the crypt-villus axis by repulsive interaction with its ligand ephrin-B1. While loss of Eph B3 is linked to colorectal cancer initiation, overexpression of Eph B3 in cancer cell lines inhibits growth and induces functional changes with decreased mesenchymal and increased epithelial markers. In order to study this tumor suppressor activity of Eph B3 in esophageal adenocarcinoma we analyzed the simultaneous expression of Eph B3 and E-cadherin in both the healthy esophagus and in Barrett’s carcinoma.

**Methods:**

Simultaneous expression of Eph B3 and E-cadherin was investigated in samples from 141 patients with Barrett’s carcinoma and from 20 healthy esophagi using immunhistology and quantitative PCR. Results from healthy squamous epithelium, Barrett’s metaplasia and staging-specific esophageal adenocarcinoma were correlated.

**Results:**

A significantly reduced E-cadherin mRNA expression could be detected in adenocarcinoma compared to dysplasia. The immunhistological activity of E-cadherin and Eph B3 was reduced in adenocarcinoma compared to dysplasia or healthy esophageal mucosa. The intracellular E-cadherin distribution changed significantly from the cytoplasm to the membrane, when the Eph receptor was simultaneously expressed. Simultaneous expression of E-cadherin and Eph B3 showed a significant inverse correlation to tumor stage.

**Conclusions:**

We present novel evidence of the tumor suppressor activity of Eph B3 in esophageal adenocarcinoma possibly due to the impact on redistribution of cellular E-cadherin to the membrane. Our results suggest that this effect might play a role in the dysplasia-adenocarcinoma sequence, the infiltrative growth pattern and the development of lymph node metastases.

## Background

Esophageal adenocarcinoma is a very aggressive cancer with a dismal five-year overall survival rate of only 20% [1-3]. These poor survival rates are due to the advanced stage of esophageal cancer at the time of diagnosis [3-5]. Even patients with early disease (for example, with a T1b-category) have lymph node metastases in up to 20% of the cases. This is known to be a strong prognostic factor for long term survival [4]. E-cadherin and Ephrin B3 receptor (Eph B3) are both transmembrane proteins that play key roles in tumorigenesis and infiltrative growth pattern with lymph node or distant metastases.

Primarily, E-cadherin and Eph B3 have been described for cell sorting, navigation and migration in embryology [6-9]. In the nervous system and the gastrointestinal tract, Ephrin receptor tyrosine kinases and their ligands, the ephrins, conduct axon guidance, development and cell intermingling [10].

In tumorigenesis of, for example, breast cancer, colorectal cancer and gastric cancer the important consequences of Eph/ephrin signaling and their interaction with E-cadherin are invasiveness, vasculature, and metastatic potential [10-15]. Two groups demonstrated an interaction of E-cadherin and Eph B3 *in vitro* in a colorectal cell line (HT-29) and a mouse model [16]. Analysis of E-cadherin and Eph B3 indicate that their coexpression suppresses cancer progression by cell-cell contacts and compartmentalization of the tumor cells. Therefore, the metastatic potential is reduced by the interaction of Eph receptors (Eph), the ephrin ligands (EFN) of the microenvironment and E-cadherin forming desmosomes for the compartmentalization of the tumor [17]. E-cadherin repression has been reported to be a late event in the sequence Barrett’s metaplasia – dysplasia – invasive carcinoma [18-20]. A correlation with cancer cell migration and an invasive growth pattern of Barrett’s carcinoma is not yet established.

1n order to investigate the possible impact of E-cadherin and Eph B3 on carcinogenesis and metastases, the expression pattern of E-cadherin and Eph B3 in patients with esophageal adenocarcinoma was analyzed with immunhistology and PCR. The results were correlated with the postoperative histopathological staging and the patients’ clinical data.

## Methods

### Patient population and study design

From February 2004 to January 2008, 141 patients with esophageal adenocarcinoma underwent surgical resection in the university hospitals of Duesseldorf (n = 61), Cologne (n = 33) and Munich (n = 47).

All patients had tumors located in the distal third of the esophagus within areas of specialized intestinal-type columnar epithelium (Barrett`s esophagus). Preoperative diagnostic work-up included gastroscopy, endosonography, biopsy of the primary tumor, computed tomography scan, and a risk assessment concerning the operability of the patient.

On primary staging 62% of the patients had an infiltration of all esophageal wall layers (uT_3_-category). Seventy-seven percent of all patients presented with enlarged and suspicious locoregional lymph nodes.

All 141 patients underwent an abdominothoracic resection. A D2 lymph node dissection was routinely done. In the chest, the lymphadenectomy included the periesophageal and infracarinal nodes. In selected patients, a lymph node dissection extending to the apex of the right chest was done. This was the case when suspicious nodes were observed in the apex during inspection. The tumors were staged according to the guidelines of the International Union against Cancer 1997.

Two biopsies of each tumor and adjacent Barrett`s mucosa were used for further examinations. All samples were snap frozen in liquid nitrogen. Samples were reviewed by an experienced pathologist. Tumor samples were estimated to contain at least 70% tumor cells with an average of 86%. For the correlation of Eph and E-cadherin and their impact on local invasiveness and lymph node or distant metastases the samples from Munich were used for immunohistochemistry (IHC) against both transmembrane proteins and Ki-67. The results were correlated with the histopathologic work-up of the surgical specimen, with staging and grading and with long-term survival. The samples from Duesseldorf and Cologne were used for RNA isolation and reverse-transcriptase polymerase chain reaction (RT-PCR) and for IHC against E-cadherin. Additional samples from healthy squamous esophageal epithelium from 20 patients with an unremarkable gastroscopy, performed for unspecific abdominal pain, were used for IHC against E-cadherin and Eph B3 for comparison. The study was approved by the Institutional Review Board. Informed consent was obtained from each patient.

### Immunohistochemistry

IHC was performed with the streptavidin-biotin system on all 141 patients and 20 control samples of healthy squamous epithelium. Snap-frozen sections were sliced at 5 μm thickness onto positively charged slides. The sections were incubated in 3% H_2_O_2_ and then blocked for unspecific binding in 1% goat serum. Then, sections were incubated in the primary monoclonal antibody overnight at 4°C (Eph B3 antibody, diluted 1:100, H2049-M01, Abnova Taiwan, Taipei; Ki-67 Mib-1 antibody, diluted 1:100, Dako, CA Carpinteria; E-cadherin, monoclonal antibody HECD-1, diluted 1:300, Takara Biomedicals, Otsu, Japan).

The IHC reactions were developed with an avidin-biotin immunoperoxidase technique (ABC method). All antibody reactions were performed in a moist chamber. Finally, the sections were immersed in hematoxylin solution for one minute. As positive controls we used sections from a healthy esophagus that was also snap frozen and treated in the same manner. Negative control reactions were carried out with an equivalently diluted mouse immunoglobulin without specific bindings and the same class of secondary antibodies.

The Ki-67 proliferation fraction represents the percentage of positively staining nuclei in each analyzed field by a minimum of 500 cells counted. For the evaluation of E-cadherin staining, more than 90%, between 10% and 90% and weak or negative staining of cells were classified as uniformly positive (2+), reduced (1+), and negative (0), respectively. The main localization of staining (cytoplasmatic versus membranous) was noted. The evaluation of Eph B3 staining was performed in the same way.

### RNA extraction and quantitative real-time reverse transcriptase PCR

Frozen tumor samples were cut in 20 μm thick sections. RNA was extracted from 10 sections by using the TRIzol reagent (Invitrogen Carlsbad, California, USA) according to the manufacturer`s instructions. The RNA concentration was verified spectrophotometrically (BioPhotomere, Eppendorf, Germany) by using the OD_260_ method. RT was performed in a volume of 20 μl by using random hexamere primer, 2 μg RNA, and transcriptor reverse transcriptase in 5x RT buffer (Roche Basel, Switzerland). PCR with cDNA was performed by using primers and probes for E-cadherin (MWG-Biotech, Ebersberg, Germany). To normalize the E-cadherin expression, we used glyceraldehyde-3-phosphate dehydrogenase (GAPDH) as the internal reference gene. For the PCR, 11.25 μl (1 ng/μl) cDNA template (or water as negative control) was mixed with 12.5 μl iQ Supermix mastermix (Bio-Rad) Munich, Bavaria, Germany and 1.25 μl primer-mix (10 μM each primer, 4 μM probe) and 76 μl H_2_O. All samples were run in duplicate. Quantitative real-time reverse transcriptase-PCR (qPCR) was carried out using the DyadDisciple Chromo 4 (Bio-Rad) with the following conditions: 95°C for 10 minutes followed by 40 cycles each comprising denaturation for 15 seconds at 95°C, annealing and extension for 1 minute at 60°C. Gene expression was quantified by determining ΔC_t_ values. The ΔC_t_ value for E-cadherin is the difference between the C_t_ for E-cadherin and for GAPDH as the internal reference control gene. High ΔC_t_ values are correlated with low levels of gene expression, whereas low ΔC_t_ values are correlated with high levels of gene expression. Since the amplification efficiencies of both genes were close to 100% (data not shown), ΔC_t_ values essentially correspond to a log-2 scale. The difference in expression between sample groups was calculated using the 2 ^-ΔΔCt^ method.

### Statistical analysis

Comparison of numerical data was done with the Student’s *t* test. Comparison of IHC results and the histologic results was done with the Fischer`s exact test and, when appropriate, with the ^2^ test. The significance of differences between groups with a non-parametric data distribution was analyzed with the Mann–Whitney *U* test for two independent groups. We used log-rank test for the univariate survival analyses. The primary endpoint was survival, as measured from first operation time to last follow-up or death. Data for patients who were still alive at the end of the study were censored. The threshold of statistical significance was set at 0.05. Statistical analysis was done using SPSS 15 for windows (SPSS, Chicago).

## Results

### Patient’s demographical data

**Figure 1:**
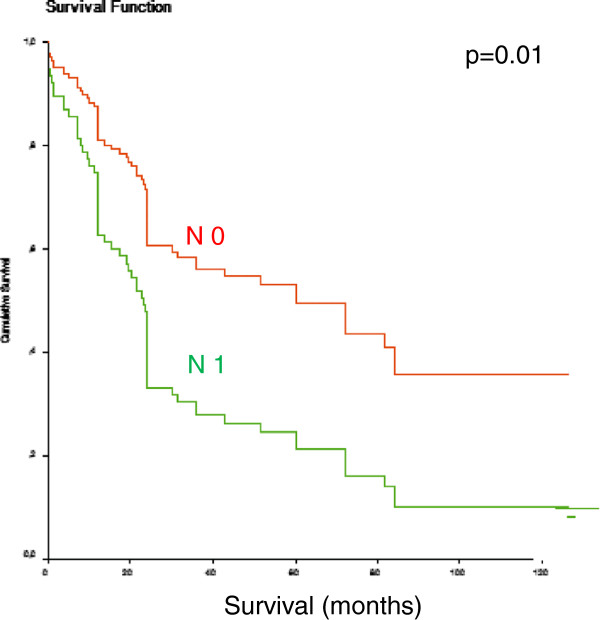
**Influence of lymph node involvement on disease related survival in patients with Barrett’s carcinoma. ***P* = 0.01.

Demographic information of the study population is summarized in Table 1. The mean age of our patients was 63.2 years ± 10.6. One hundred sixteen patients were men, 25 were women. The final histologic work-up of the surgical specimens is presented in Table 1. In most cases all wall layers were involved (T_3_-category) and positive lymph nodes could be found (61.7%). In 11 patients initially unknown distant metastases could histologically be proven intraoperatively. Five liver metastases, three pulmonary metastases and three infiltrated paraaortic lymph nodes, counting as distant metastases, were detected. All metastases were completely removed. Median survival was 27 months in all patients. Lymph node involvement was a strong predictive factor in our patients. In patients without lymph node metastases median survival rose to 74 months (*P* = 0.01) (Figure 1).

**Table 1:** Demographic data and correlation of tumor characteristics in patients with positive versus negative E-cadherin immunhistology expression

	**Patients**	**E-cadherin IHC expression**		***P*-value**
	n = 141	positive (n = 79)	negative (n = 62)	
Gender (M/F)	116/ 25	63/ 16	53/ 9	0.5
Patient median age (y) (range)	64 (36 – 84)	63 (36–82)	64 (39–84)	0.276
Depth of invasion:				0.218
pT_0_	5	4	1	
pT_1_	16	12	4	
pT_2_	58	29	29	
pT_3_	62	34	28	
Lymph node metastasis:				0.117
pN_0_	54	35	19	
pN_1_	87	44	43	
Metastases:				0.535
M_0_	130	74	56	
M_1_	11	5	6	
Grading:				0.307
G1	2	2	0	
G2	38	25	13	
G3	84	43	41	
G4	17	9	8	

*F* female, *IHC* immunhistology, *M* male, *y* year.

### Immunohistochemical analysis of E-cadherin, Eph B3 and ki-67

First the E-cadherin immunostaining was performed in the normal squamous epithelium of the esophagus. In almost 100% of all investigated cases a strong staining of the cell membrane could be detected. This strong membranous staining was categorized as 2+. The E-cadherin staining in the 141 tumor samples was only positive in 56% of cases. An equally strong membranous staining (2+) could only be found in 21% of the cases. There was no significant correlation comparing the strength of the E-cadherin immunhistology with the histopathological staging, especially the lymph node involvement (Tables 1 and 2)

**Table 2:** Correlation of lymph node status and E-cadherin immunhistology expression differentiated according to the expression rate

		**N-status**		
		N_0_	N_1_	
	0	19	43	62
E-cadherin-	1+	24	26	50
expression	2+	11	18	29
		54	87	141

*P* = 0.179. *N-status* lymph node status.

**Figure 2:**
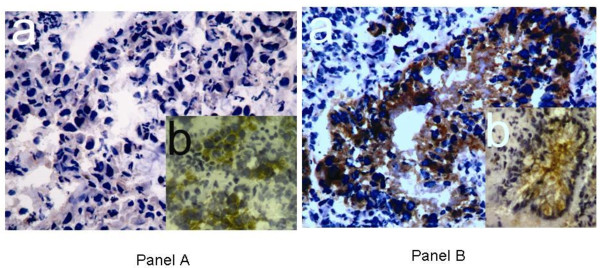
**Panel A: Anti-Eph B3 immunhistology (a) and anti-E-cadherin immunhistology (b) in a patient with a poorly differentiated esophageal adenocarcinoma.** Eph B3 immunhistology is negative. The E-cadherin immunhistology shows a faint cytoplasmatic reaction. 40-fold magnification. **Panel B**: Anti-Eph B3 immunhistology (**a**) and anti-E-cadherin immunhistology (**b**) in a patient with a moderately differentiated esophageal adenocarcinoma. The Eph B3 shows a strong positivity. The E-cadherin shows a moderate (1+) reaction of the tumor cell membrane. 40-fold magnification. Eph B3, ephrin B3.

**Figure 3:**
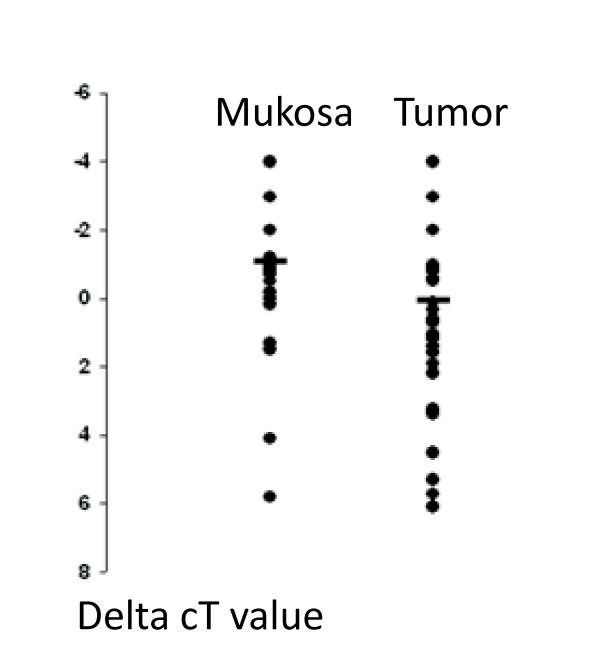
**Real-time PCR analyses of Barrett metaplasias (mucosa) and the adjacent Barrett carcinomas (tumor).** C_t_ values were determined from duplicate reactions. ΔC_t_ were obtained by subtracting the mean C_t_ value for GAPDH from the mean C_t_ value from E-cadherin. A lower ΔC_t_ value indicates a higher gene expression. The horizontal bars indicate the mean values. The E-cadherin expression level was significantly different between the two groups (*P* = 0,02, Mann_Whitney *U* Test). GAPDH, glyceraldehyde-3-phosphate dehydrogenase.

Interestingly, the intracellular distribution of E-cadherin changed between normal mucosa compared to tumor samples. In tumor samples the membrane-bound E-cadherin strongly decreased, while cytoplasmatic E-cadherin showed an increasing immunohistologic reaction at the same time (Figures 2 and 3). A strong membranous immunohistological reaction could be found in less than 10% of cases.

The Eph B3 IHC showed a significant correlation with the E-cadherin distribution in tumor cells. In patients with a coexpression in both IHC, the E-cadherin showed a strong membranous accentuation without a cytoplasmatic reaction (*P* = 0.003) (Figure 2).

The E-cadherin/Eph B3 immunhistology coexpression was compared to the pathological staging of the postoperative specimen. Even moderate (1+) immunohistological coexpression was significantly correlated with depth of invasion and lymph node involvement (Table 3). The lymph node coefficient (= lymph node metastases/number of removed lymph nodes), which reflects a significant calculated prognostic factor for long-term survival of patients with esophageal adenocarcinoma, also significantly depends on the coexpression of E-cadherin/Eph B3 (*P* = 0.017) (Table 3).

**Table 3:** Demographic data and correlation of tumor characteristics in patients with positive versus negative simultaneous Eph B3 and E-cadherin immunhistology expression

	**E-cadherin/Eph B3 - Coexpression**		***P*-value**
	positive (n = 16)	negative (n = 31)	
Gender (M/F)	15/ 1	24/ 7	0.234
Patient median age (y) (range)	56 (41–73)	63 (44–84)	0.069
Depth of invasion:			0.023
pT_0_	4	1	
pT_1_	5	5	
pT_2_	3	5	
pT_3_	4	20	
Lymph node metastasis:			0.001
pN_0_	16	9	
pN_1_	0	22	
Lymph node coefficient:			0.017
>0.1	1	10	
<0.1	15	21	
Resection status:			0.472
R0	16	26	
R1	0	5	
Grading:			0.182
G1	2	0	
G2	4	9	
G3	10	22	

*Eph B3* ephrin B3, *F* female, *M* male, *y* years.

The Ki-67 IHC had a significantly higher activity in tumor samples compared to the Barrett`s epithelium (*P* = 0.001). In the dysplastic epithelium 23.7 ± 9% of cells were positive, whereas we observed a significantly higher Ki-67 proliferation fraction with 36.7 ± 12% positive cells in the tumor samples. The Ki-67 activity did not affect the histopathological staging or grading.

### Quantitative real-time RT-PCR analysis

In order to validate our immunohistological findings on a RNA basis and in order to analyze the E-cadherin mRNA expression in Barrett`s metaplasia and adjacent carcinoma, we performed a qPCR and correlated the results with the histopathological data calculated using the 2 ^-ΔΔCt^ method. A significant decrease of E-cadherin mRNA expression could be detected when comparing the metaplasia and the neighboring adenocarcinoma (*P* = 0.02) (Figure 3). Corresponding to the immunohistological results, differences of the E-cadherin mRNA expression rate in the tumor in correlation with the histopathological work-up (depth of invasion, lymph node status, distant metastases, grading) or clinical course (disease free survival) could not be found.

### The E-cadherin mRNA expression rate was not correlated with the age or sex of the patients

## Discussion

The only generally accepted marker for increased risk of esophageal adenocarcinoma is the presence of high-grade dysplasia (HGD) [21]. Once a carcinoma has developed, lymph node metastasis can occur in the very early stages [4]. According to the literature, lymph node metastases can be found in approximately 25% of patients with a T_1_-category. Lymph node metastases are a strong predictor for a dismal long-term survival, which is reflected in our survival analysis with a five-year survival rate of patients with involved lymph nodes of <30% compared to patients without lymph node metastasis of >50% (*P* = 0.01). In order to characterize specific molecular changes between Barrett’s epithelium and esophageal adenocarcinoma and the changes in higher tumor stages with lymph node involvement, we analyzed E-cadherin and Eph B3 in 141 patients with esophageal adenocarcinoma.

E-cadherin is a calcium dependent transmembrane adhesion protein [22]. During embryonic development, E-cadherin is expressed in all cells, but it disappears in embryonic mesoderm cells [23]. Epithelial-to-mesenchymal transformation is a process by which polarized epithelial cells convert to non-polarized and motile cells to allow histogenesis, organogenesis and morphogenesis in embryonic development [24]. Epithelial tumors implement a similar process known as epithelial-to-mesenchymal transition (EMT), during dedifferentiation, invasion and metastasis in the process of tumor progression [25,26]. Lack of E-cadherin-mediated adhesion in human tumors correlates with loss of epithelial morphology and acquisition of mesenchymal invasive characteristics. E-cadherin is believed to be the master programmer of EMT, because signaling pathways contributing to EMT converge on regulation of this molecule [27]. In particular, E-cadherin has been implicated in the malignant progression of Barrett’s metaplasia to adenocarcinoma [19,28-30]. In our patient group we show by quantitative PCR a significant reduction of E-cadherin expression in adenocarcinoma compared to Barrett’s metaplasia. This difference in mRNA expression could be clearly reconfirmed on the protein level with IHC in our patients. Corresponding to the international literature, a correlation could not be found between the E-cadherin PCR and the strength of IHC reactivity and the staging of the carcinoma [19,20]. In order to further elucidate the tumor progression Eph B3 was analyzed in our patients.

In the mammalian gut, Eph B3 is expressed in cells at the bottom of the intestinal crypts near stem cell niches [31,32]. Physiologically, Eph B3 is responsible for constructing and maintaining the architecture of the crypt-villus axis in the intestinal epithelium by repulsive interaction with its ligand (EFN B1) [31]. A characteristic of the Eph B/EFN B signaling system is the ability to elicit bi-directional signaling that leads to the restriction of cell migration and cell intermingling across segmental boundaries [32]. Pathologically, loss of the Eph B3 allele induces the development of colon adenomas as the first morphological step towards colon cancer, whereas Eph B-mediated compartmentalization could be demonstrated to be a mechanism suppressing colorectal cancer progression [16,17,33]. These findings suggest that Eph B3 functions as a tumor suppressor. Analogous to the findings in colon cancer, in our patients Eph B3/E-cadherin coexpression is significantly correlated with a favorable tumor stage. Although E-cadherin mRNA expression and IHC positivity did not show a significant difference with regard to tumor stage, coexpression of both proteins was significantly and inversely correlated with depth of invasion, lymph node involvement and lymph node coefficient (= number of involved lymph nodes/number of removed lymph nodes).

Recently *in vitro* analysis indicated that Eph B activation triggered redistribution of E-cadherin from the cytoplasm to the basolateral membrane without altering protein levels in a colon cancer cell line. Consequently, Eph B signaling couples cell contraction with cell-to-cell-adhesion by promoting the recruitment of E-cadherin in colon cancer [17]. This mechanism may be equally present in esophageal cancer. In our study, simultaneous expression of E-cadherin and Eph B3 was accompanied by an intracellular E-cadherin distribution comparable to that in healthy esophageal mucosal cells. A strong membranous accentuated immunohistologic reaction was seen in 10% of all patients, while we detected a fairly strong cytoplasmatic and a faint membranous staining in carcinoma cells without Eph B3 expression.

Taking the *in vitro* analysis by Cortina into account, the above observations suggest that Eph B signaling seems to restrict the capacity of malignant cells for infiltrative growth by enforcing E-cadherin adhesion. In an *Apc*^Min/+^ mouse model the EphB mediated compartmentalization was demonstrated to be a mechanism suppressing cancer progression [17]. In a clinical study Eph B3 expression was significantly reduced in advanced Dukes’ stage tumor specimens [16]. *In vitro* examinations of a colon cancer cell line (HT-29) demonstrated that Eph B3/EFN interaction potentiated junctional adhesion molecules ZO-1, E-cadherin and plakoglobin, which are representatives of tight junctions and desmosomes, respectively [16].

In regard to the literature concerning the interaction between Eph B3 and E-cadherin, both proteins together have a significant tumor suppressor function. Comparable to the tumorigenesis of colon cancer we could show an altered Eph B3 and E-cadherin IHC activity of esophageal carcinoma compared to the normal mucosa and a reduced E-cadherin mRNA expression rate in esophageal carcinoma compared to normal mucosa. In the dysplasia-carcinoma sequence Eph B3 activity is reduced and E-cadherin is dissolved in the cytoplasm. Lack of E-cadherin-mediated adhesion correlates with the loss of epithelial morphology and the acquisition of mesenchymal characteristics. In our patients with esophageal cancer we could find a significant inverse correlation between a persisting simultaneous expression of Eph B3 and E-cadherin and depth of invasion and lymph node metastasis as the strongest predictive factors for long-term survival. Corresponding to colon cancer we assume from our findings an impact of Eph B3 on E-cadherin and reinforcement of the cell-cell-junctions in esophageal cancer.

## Conclusions

We demonstrated an association of the Eph B3/E-cadherin coexpression with the metaplasia adenocarcinoma sequence in Barrett’s carcinoma for the first time on protein and RNA bases. Migration and metastatic potential also seems to be influenced by the interaction of these proteins. The rare persisting simultaneous expression of E-cadherin and Eph B3 in esophageal adenocarcinoma is intimately associated with an early favorable tumor stage. The simultaneous expression of E-cadherin and Eph B3 has the potential to serve as a new biological marker for the characterization of the individual tumor biology with regard to local invasion and lymph node involvement. The direct interaction in terms of EMT and metastatic potential has to be further investigated in an animal model.

## Abbreviations

EFN, Ephrin ligands; EMT, Epithelial-to-mesenchymal transition; Eph, Ephrin; IHC, Immunohistochemistry; RNA, Ribonucleic acid; PCR, Polymerase chain reaction, M, Male; y, Years; HGD, High grade dysplasie.

## **Competing interests**

There are no competing interests for all authors.

## **Authors’ contributions**

MCS participated in the design of the study and performed the statistical analysis. MCS, NS and SB carried out the laboratory tests. JT, FK, AH, MF, EB, CM, WB and WTK helped to design and draft the manuscript. All authors read and approved the final manuscript.

## References

[B1] Bonavina L Via A Incarbone R Saino G Peracchia A Results of surgical therapy in patients with Barrett`s adenocarcinoma World J Surg 2003 27 1062 1066 10.1007/s00268-003-7062-0 12917757

[B2] Fountoulakis A Zafirellis D Dolan K Dexter SP Martin IG Sue-Ling HM Effect of surveillance of Barrett`s oesophagus on the clinical outcome of oesophageal cancer Br J Surg 2004 91 997 1003 10.1002/bjs.4591 15286961

[B3] Shaheen N Advances in Barrett`s esophageal adenocarcinoma Gastroenterology 2005 128 1554 1566 10.1053/j.gastro.2005.03.032 15887151

[B4] Feith M Stein H Siewert R Pattern of lymphatic spread of Barrett`s cancer World J Surg 2003 27 1052 1057 10.1007/s00268-003-7060-2 12917758

[B5] Siewert J Stein H Feith M Surgical approach to invasive adenocarcinoma of the distal esophagus (Barrett`s cancer) World J Surg 2003 27 1058 1061 10.1007/s00268-003-7061-1 12925905

[B6] Gale NM Holland SJ Valenzuenla DM Eph receptors and ligands comprise two major specificity subclasses, and are reciprocally compartmentalized during embryogenesis Neuron 1996 17 9 19 10.1016/S0896-6273(00)80276-7 8755474

[B7] Mellitzer G Xu Q Wilkinson DG Eph receptors and ephrins restrict cell intermingling and communication Nature 1999 400 77 81 10.1038/21907 10403252

[B8] Wang HU Anderson DJ Eph family transmembrane ligands can mediate repulsive guidance of trunk neural crest migration and motor axon outgrowth Neuron 1997 18 383 396 10.1016/S0896-6273(00)81240-4 9115733

[B9] Xu Q Mellitzer R Robinson V Wilkinson DG In vivo cell sorting in complementary segmental domains mediated by ephrin receptors and ephrins Nature 1999 399 267 271 10.1038/20452 10353250

[B10] Katoh Y Katoh M Comparative integromics on ephrin family Oncol Rep 2006 15 1391 1395 16596216

[B11] Birchmeier W Behrens J Cadherin expression in carcinomas: role in the formation of cell junctions and the prevention of invasiveness Biochim Biophys Acta 1994 1198 11 26 8199193 10.1016/0304-419x(94)90003-5

[B12] Brantley-Sieders D Schmidt S Parker M Chen J Eph receptor tyrosine kinases in tumor and tumor microenvironment Curr Pharm Des 2004 10 3431 3442 10.2174/1381612043383160 15544526

[B13] Debruyne P Vermeulen S Mareel M The role of E-cadherin/catenin complex in gastrointestinal cancer Acta Gastroenterol Belg 1999 62 393 402 10692769

[B14] Nollet F Berx G van Roy F The role of the E-cadherin/catenin adhesion complex in the development and progression of cancer Mol Cell Biol Res Commun 1999 2 77 85 10.1006/mcbr.1999.0155 10542129

[B15] Surawska H Ma PC Salgia R The role of ephrins and Eph receptors in cancer Cytokine Growth Factor Rev 2004 15 419 433 10.1016/j.cytogfr.2004.09.002 15561600

[B16] Chiu ST Chang KJ Ting CH Shen HC Li H Hsieh FJ Over-expression of EphB3 enhances cell-cell contacts and suppresses tumor growth in HT-29 human colon cancer cells Carcinogenesis 2009 30 1475 1486 10.1093/carcin/bgp133 19483190

[B17] Cortina C Palomo-Ponce S Iglesias M Fernández-Masip JL Vivancos A Whissell G Humà M Peiró N Gallego L Jonkheer S Davy A Lloreta J Sancho E Batlle E EphB-ephrin-B interactions suppress colorectal cancer progression by compartmentalizing tumor cells Nat Genet 2007 39 1376 1383 10.1038/ng.2007.11 17906625

[B18] Falkenback D Nilbert M Oberg S Johansson J Prognostic value of cell adhesion in esophageal adenocarcinomas Dis Esophagus 2008 21 97 102 10.1111/j.1442-2050.2007.00749.x 18269642

[B19] Feith M Stein HJ Mueller J Siewert JR Malignant degeneration of Barrett’s esophagus: the role of the Ki-67 proliferation fraction, expression of E-cadherin and p53 Dis Esophagus 2004 17 322 327 10.1111/j.1442-2050.2004.00434.x 15569371

[B20] Swami S Kumble G Striadafilopoulos G E-cadherin expression in gastroesophageal reflux disease, Barrett’s esophagus, and esophageal adenocarcinoma An immunhistochemical immunoblot study. Am J Gastroenterol 1995 90 1808 1813 7572899

[B21] Flejou JF Svrcek M Barrett’s esophagus – a pathologist’s view Histopathology 2007 50 3 14 10.1111/j.1365-2559.2006.02569.x 17204017

[B22] Gumbiner B Stevenson B Grimaldi A The role of the cell adhesion molecule uvomorulin in the formation and maintenance of the epithelial junctional complex J Cell Biol 1988 107 1575 1587 10.1083/jcb.107.4.1575 3049625 PMC2115263

[B23] Butz S Larue L Expression of catenins during mouse embryonic development and in adult tissues Cell Adhes Commun 1995 3 337 352 10.3109/15419069509081018 8821035

[B24] Hay ED The mesenchymal cell, its role in the embryo, and the remarkable signalling mechanisms that create it Dev Dyn 2005 233 706 720 10.1002/dvdy.20345 15937929

[B25] Gavert N Ben-Ze’ev A Epithelial-mesenchymal transition and the invasive potential of tumors Trends Mol Med 2008 14 199 209 10.1016/j.molmed.2008.03.004 18406208

[B26] Lee JM Dedhar S Kalluri R Thompson EW The epithelial-mesenchymal transition: new insights in signalling, development, and disease J Cell Biol 2006 27 973 981 16567498 10.1083/jcb.200601018 PMC2063755

[B27] Guarino M Rubino B Ballabio G The role of epithelial-mesenchymal transition in cancer pathology Pathology 2007 39 305 318 10.1080/00313020701329914 17558857

[B28] Helm J Enkemann S Coppola D Barthel JS Kelley ST Yeatman TJ Dedifferentiation precedes invasion in the progression from Barrett`s metaplasia to esophageal adenocarcinoma Clin Cancer Res 2005 11 2478 2485 10.1158/1078-0432.CCR-04-1280 15814623

[B29] Darlavoix T Seelentag W Yan P Bachmann A Bosman FT Altered expression of CD44 and DKK1 in the progression of Barrett’s esophagus to esophageal adenocarcinoma Virchows Arch 2009 454 629 637 10.1007/s00428-009-0769-z 19396460

[B30] Reid BJ Levine DS Longton G Blount PL Rabinovitch PS Predictors of progression to cancer in Barrett’s esophagus: baseline histology and flow cytometry identify low- and high-risk patient subsets Am J Gastroenterol 2000 95 1669 1676 10925966 10.1111/j.1572-0241.2000.02196.x PMC1783835

[B31] Batlle E Henderson J Beghtel H Born M Sancho E Huls G Meeldijk J Robertson J Wetering M Pawson T Clevers H β-catenin and TCF mediate cell positioning in the intestinal epithelium by controlling the expression of EphB/EphrinB Cell 2002 111 251 263 10.1016/S0092-8674(02)01015-2 12408869

[B32] Holmberg J Genander M Halford MM Anneren C Sondell M Chumley MJ Silvany RE Henkemeyer M Frisen J EphB receptors coordinate migration and proliferation in the intestinal stem cell niche Cell 2006 125 1151 1163 10.1016/j.cell.2006.04.030 16777604

[B33] Batlle E Bacani J Begthel H Jonkheer S Gregorieff A van de Born M Malats M Sancho E Boon E Pawson T Gallinger S Pals S Clevers H EphB receptor activity suppresses colorectal cancer progression Nature 2005 435 1126 1130 Additional Query: May 3, 2012 10.1038/nature03626 15973414

